# Brexpiprazole's impacts on patients and caregivers in agitation in Alzheimer's dementia

**DOI:** 10.1002/alz.70522

**Published:** 2025-07-28

**Authors:** Yu Nakamura, Jun Adachi, Naoki Hirota, Katsuhiro Iba, Cosmo Sasajima, Koichi Shimizu, Masami Nakai, Kaneyoshi Takahashi

**Affiliations:** ^1^ Faculty of Medicine/Graduate School of Medicine Kagawa University Kagawa Japan; ^2^ Headquarters of Clinical Development Otsuka Pharmaceutical Co., Ltd Tokyo Japan; ^3^ Headquarters of Clinical Development Otsuka Pharmaceutical Co., Ltd Osaka Japan; ^4^ Department of Medical Affairs Otsuka Pharmaceutical Co., Ltd Tokyo Japan; ^5^ Department of Medical Affairs Otsuka Pharmaceutical Co., Ltd Osaka Japan

**Keywords:** agitation, Alzheimer's dementia, behavioral and psychological symptoms of dementia, brexpiprazole, caregivers, Japan, Neuropsychiatric Inventory

## Abstract

**INTRODUCTION:**

We evaluated the impacts of brexpiprazole treatment on patient neuropsychiatric symptoms and caregiver distress in Japanese patients with agitation in Alzheimer's dementia (AAD).

**METHODS:**

In a phase 2/3 multicenter, double‐blind study, patients were randomized to receive brexpiprazole 1 or 2 mg/day, or placebo (3:4:4) for 10 weeks. The Neuropsychiatric Inventory (NPI) was used for evaluation. The areas of patient symptoms and caregiver distress were defined as NPI and NPI‐Distress, respectively.

**RESULTS:**

At Week 10, the differences of brexpiprazole 1 and 2 mg versus placebo for NPI total score were −1.2 (*p* = 0.5891) and −8.4 (*p* < 0.0001), and those for NPI‐Distress total score were −1.1 (*p* = 0.2292) and −3.9 (*p* < 0.0001), respectively. In brexpiprazole 2 mg versus placebo, NPI and NPI‐Distress agitation/aggression score showed ≥ 2 and ≥ 1 point improvement, respectively.

**DISCUSSION:**

Brexpiprazole is suggested to improve patient symptoms and to reduce caregiver distress in the treatment of AAD in Japanese patients.

**TRIAL REGISTRATION:**

ClinicalTrials.gov Identifier NCT03620981

**Highlights:**

- Brexpiprazole improved symptoms of patients with agitation in Alzheimer's dementia.
- The improvements included not only agitation, but also various behavioral and psychological symptoms of dementia scores.
- The improvements of patient symptoms led to the reduction of caregiver distress.
- Our study suggests that brexpiprazole is beneficial for both patients and caregivers.

## 1 BACKGROUND

The number of patients with dementia is increasing and projected to be 139 million by 2050, which is almost twice as many as 2024.^1^ Patients with dementia suffer from cognitive decline and behavioral disturbances,^2^, ^3^ being a source of social disadvantage and exclusion.^4^ Dementia also imposes an economic burden on society and families, and causes increases in health‐care costs and the loss of productivity.^5^, ^6^ These reports suggest that dementia is related to various problems.

Of all types of dementia, Alzheimer's dementia is the most common type.^5^, ^7^ Its symptoms consist of cognitive dysfunction, and accompanying symptoms called behavioral and psychological symptoms of dementia (BPSD).^8^ Agitation is one of the BPSD,^9^, ^10^, ^11^ and is observed in approximately half of patients with Alzheimer's disease/dementia.^12^, ^13^ Agitation is suggested to increase caregiver distress, which can lead to burnout or potentially harmful behavior of caregivers.^14^, ^15^ Physical aggression (one form of agitation) is associated with institutionalization of patients with Alzheimer's disease.^16^ These reports show that agitation affects both patients and caregivers, and its burden is therefore worth treating independently of cognitive dysfunction in Alzheimer's disease.

Brexpiprazole is the first US Food and Drug Administration–approved drug indicated for agitation in Azheimer's dementia (AAD). As of February 2025, brexpiprazole for this indication is approved only in a limited number of countries, that is, the United States, Canada, the Philippines, Taiwan, Thailand, Turkey, Malaysia, Singapore, Australia, and Japan. Brexpiprazole is an antipsychotic that acts as a partial agonist at serotonin 5‐hydroxytryptamine_1A_ and dopamine D_2_ receptors, and at the same time functions as an antagonist at serotonin 5‐hydroxytryptamine_2A_ and noradrenaline α_1B_/α_2C_ receptors.^17^, ^18^ These neurotransmitters like serotonin, dopamine, and noradrenaline are considered related to agitation.^19^

**RESEARCH IN CONTEXT**

1. **Systematic review**: For the treatment of agitation in Alzheimer's dementia, brexpiprazole is approved in a limited number of countries, and its clinical profile is still evolving globally. After the publication of a phase 3 confirmation study by Nakamura et al., we further analyzed the exploratory endpoints (various Neuropsychiatric Inventory scores) to study the benefit of brexpiprazole in both patients and caregivers.
2. **Interpretation**: Brexpiprazole treatment improved patient neuropsychiatric symptoms, which included not only agitation, but also various behavioral and psychological symptoms of dementia items. These results supported the result of the primary endpoint reported previously.^20^ The improvements of patient symptoms led to the reduction of caregiver distress, suggesting that brexpiprazole is beneficial for both patients and caregivers.
3. **Future directions**: As the treatment period of this study was 10 weeks, further data collection for a longer term is expected. The recurrence of symptoms and the impact on caregivers after the cessation of brexpiprazole treatment also remain to be studied.

Brexpiprazole has demonstrated efficacy and safety in a pivotal phase 2/3 study (randomized, double‐blind, placebo‐controlled, 10‐week treatment with brexpiprazole 1 or 2 mg/day),^20^ which is the basis of the regulatory approval of brexpiprazole indicated for AAD in Japan. In the extension study, eligible patients were rolled over, and the extended 14 week treatment was generally well tolerated with the efficacy maintained.^21^ Similarly, the short‐term and long‐term efficacy and safety of brexpiprazole treatment for AAD in the international studies have also been reported.^22^ ^–^ ^24^ Based on two of three international pivotal studies, the results of a post hoc pooled analysis with Neuropsychiatric Inventory (NPI) were presented at the American Association for Geriatric Psychiatry 2024 annual meeting, which suggested the efficacy of brexpiprazole across agitation behaviors, including impact on caregivers.^25^ However, these data do not represent the population in the pivotal study conducted in Japan.

The Cohen–Mansfield Agitation Inventory (CMAI), the primary endpoint in the pivotal study in Japan, is a clinically validated scale that quantifies the frequency of 29 agitated behaviors during a period of 2 weeks^26^, ^27^, ^28^ that is commonly used in clinical trials, but not much used in clinical practice in real‐world settings and does not evaluate the severity of agitation. The exploratory endpoints in this pivotal study included NPI and the NPI Nursing Home Version (NPI‐NH), which are questionnaires to evaluate BPSD.^29^, ^30^ These questionnaires are recommended in Clinical Practice Guideline for Dementia 2017,^8^ and are actually used in the clinical practice globally.^31^ The scores of NPI (for patients at home) and NPI‐NH (for patients in a hospital or care facility) are calculated by multiplying frequency and severity scores, and thus can evaluate symptoms with low frequency and high severity, which makes it difficult for caregivers to care for patients, even if frequency is low. NPI and NPI‐NH can also quantify caregiver distress and occupational disruptiveness, respectively.^32^, ^33^, ^34^

The purpose of these exploratory analyses was, using NPI and NPI‐NH, to evaluate BPSD symptoms in brexpiprazole‐treated patients with AAD and its impact on caregiver distress, which will provide useful data for better understanding of patient symptoms and caregiver distress.

## 2 METHODS

### 2.1 Study design

The detailed methodology has previously been reported.^20^ Briefly, for the key inclusion criteria, patients had to be 55 to 90 years old and must have had diagnoses of Alzheimer's dementia by Diagnostic and Statistical Manual of Mental Disorders, Fifth Edition (DSM‐5), and probable Alzheimer's disease by the National Institute of Neurological and Communicative Disorders and Stroke–Alzheimer's Disease and Related Disorders Association.^35^, ^36^ Patients also must have had a score of ≥ 4 in the agitation/aggression domain score (frequency × severity) of NPI or NPI‐NH at the screening test and the baseline assessment. For the key exclusion criteria, patients with dementia other than Alzheimer's type or memory impairment were excluded. Patients who had delirium, schizophrenia spectrum, other psychotic disorders, bipolar disorder, bipolar‐related disorders, and major depressive disorder, according to DSM‐5, were also excluded. Eligible patients were randomized in a 3:4:4 ratio to brexpiprazole 1 or 2 mg/day, or placebo, for 10 weeks.

This study was registered in ClinicalTrials.gov (NCT03620981) and conducted in compliance with the International Council on Harmonisation Good Clinical Practice and local regulations. The protocol, its amendments, and informed consent forms were reviewed and approved by each site's institutional review boards. Written informed consent was obtained from patients and/or their legal representatives and caregivers after the procedures had been fully explained. The study was conducted at 120 sites nationwide in Japan, including not only large hospitals but also small clinics, with diversity, equity, and inclusion in consideration.

### 2.2 Assessments

NPI and NPI‐NH were used to evaluate the 12 domains of BPSD by ratings of a caregiver. The 12 domains were as follows: delusions, hallucinations, agitation/aggression, depression/dysphoria, anxiety, elation/euphoria, apathy/indifference, disinhibition, irritability/lability, aberrant motor behavior, sleep and nighttime behavior disorders, and appetite and eating disorders. Each domain was scored by a 4 point scale of frequency (1: rarely, 2: sometimes, 3: often, 4: very often) multiplied by a 3 point scale of severity (1: mild, 2: moderate, 3: severe). NPI and NPI‐NH also included questions to evaluate how much distress each symptom caused a caregiver. Each domain was scored by a 6 point scale of caregiver distress and occupational disruptiveness, respectively (0: not at all, 1: minimally, 2: mildly, 3: moderately, 4: severely, 5: very severely or extremely). NPI was used for patients at home, whereas NPI‐NH was used for patients in a hospital or care facility. Hereafter, NPI and NPI‐NH were combined into and referred to as NPI. The areas of patient symptoms and caregiver distress/disruptiveness were distinguished with or without “‐Distress”; that is, NPI and NPI‐Distress, respectively. A decrease of score means an improvement of patient symptoms or a reduction of caregiver distress. These scores were assessed at baseline, Week 4, and Week 10. It should be noted that each domain of the NPI individual scores and NPI‐Distress individual scores was 12 points and 5 points at a maximum, respectively, and that the maximum scores of NPI total score and NPI‐Distress total score were 144 points (12 points × 12 domains) and 60 points (5 points × 12 domains), respectively. These settings mean that room for improvement was smaller in the NPI‐Distress score than in the NPI score.

According to the overview of the 2020 patient survey by Japan's Ministry of Health, Labor and Welfare, the average length of hospitalization of patients with Alzheimer's disease was 273 days.^37^ Our treatment/observation period of 10 weeks was within the range of this period.

### 2.3 Statistical analysis

The analyses of NPI and NPI‐Distress were performed by mixed models for repeated measures (MMRM), based on the assumption of missing at random, using an observed cases dataset. The model included, as factors, treatment group (brexpiprazole 1 mg, 2 mg, or placebo), visit (Week 4 or Week 10), medical care category (inpatient or outpatient [home or care facility]), prior use of antipsychotics (yes or no), and interaction between treatment group and visit, and as covariates, baseline and interaction between baseline and visit (the repeated measures effects were modeled as an unstructured error covariance structure). The model did not include age and sex as factors or covariates, and did not differentiate home and care facility within the category of outpatients. The Kenward–Roger method was used to approximate degrees of freedom. For the primary efficacy endpoint (CMAI) reported previously,^20^ multiplicity was adjusted to control type I error and statistical power was considered to control type II error, but not for the exploratory endpoints (NPI and NPI‐Distress) reported in this article. For standardized mean difference (SMD), Cohen's d was calculated. The full analysis set consisted of patients who were randomized, administered at least one dose of study medication, and had CMAI total score at baseline and at least one occasion after baseline.

## 3 RESULTS

### 3.1 Patients

As reported previously,^20^ 410 eligible patients were randomized at 3:4:4 ratio to brexpiprazole 1 or 2 mg, or placebo, that is, 112, 149, and 149 patients, respectively. All randomized patients received study medication at least once, and the study completion rates were 74.1% (83/112), 68.5% (102/149), and 77.9% (116/149), for 1 mg, 2 mg, and placebo, respectively.

The demographic and clinical characteristics at baseline were similar across treatment groups.^20^ In total, the mean (standard deviation [SD]) age was 80.1 (6.7) years and the percentage of female patients was 63.8% (as a side note, sex distribution was a little uneven: the percentage of female patients in the placebo, brexpiprazole 1 mg, and 2 mg groups was 57.8%, 62.0%, and 70.9%, respectively). In terms of medical care category, approximately 70% of patients were in a hospital, > 20% were at home, and < 10% were in a care facility. Clinical status of patients by hospital, and home or care facility was similar across treatment groups (Table 1).

**TABLE 1:** Clinical status of patients by hospital, and home or care facility (FAS).

Characteristics	Placebo	Brex 1 mg	Brex 2 mg	Total Brex	Total
**Overall**					
CMAI total score					
Number of patients	147	108	148	256	403
Mean (SD)	62.7 (11.7)	62.1 (11.3)	64.1 (12.9)	63.3 (12.3)	63.1 (12.0)
Alzheimer's disease (months)^*^					
Number of patients	143	105	143	248	391
Mean (SD)	61.2 (39.9)	65.6 (45.1)	66.0 (40.0)	65.8 (42.1)	64.1 (41.3)
Agitation From Alzheimer's disease (months)^*^					
Number of patients	146	108	146	254	400
Mean (SD)	25.7 (27.2)	22.7 (23.2)	25.1 (25.2)	24.1 (24.4)	24.7 (25.4)
**Hospital**					
CMAI total score					
Number of patients	102	75	102	177	279
Mean (SD)	61.1 (10.4)	63.3 (11.2)	63.1 (12.5)	63.2 (12.0)	62.4 (11.5)
Alzheimer's disease (months)^*^					
Number of patients	98	72	98	170	268
Mean (SD)	64.0 (43.3)	64.7 (45.6)	67.4 (39.0)	66.2 (41.8)	65.4 (42.3)
Agitation From Alzheimer's disease (months)^*^					
Number of patients	101	75	100	175	276
Mean (SD)	23.9 (26.7)	21.5 (20.7)	23.0 (23.1)	22.3 (22.0)	22.9 (23.8)
**Home or care facility**					
CMAI total score					
Number of patients	45	33	46	79	124
Mean (SD)	66.3 (13.6)	59.3 (11.2)	66.4 (13.5)	63.4 (13.0)	64.5 (13.2)
Alzheimer's disease (months)^*^					
Number of patients	45	33	45	78	123
Mean (SD)	55.3 (30.7)	67.6 (44.8)	62.8 (42.3)	64.8 (43.1)	61.3 (39.2)
Agitation From Alzheimer's disease (months)^*^					
Number of patients	45	33	46	79	124
Mean (SD)	29.9 (28.3)	25.5 (28.2)	29.8 (29.1)	28.0 (28.6)	28.7 (28.4)

Abbreviations: Brex, brexpiprazole; CMAI, Cohen–Mansfield Agitation Inventory; FAS, full analysis set; SD, standard deviation.

* Months derived based on (date of assessment ‐ estimated date of onset + 1)/30. Any unknown month or day of onset is imputed with June or 15, respectively.

### 3.2 Efficacy

**FIGURE 1:**
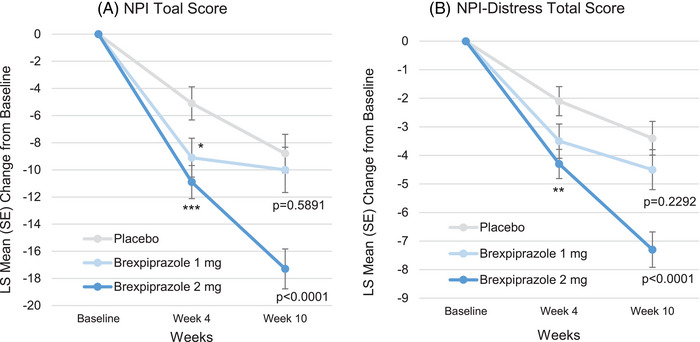
Time course of NPI and NPI‐Distress total scores (FAS). MMRM analysis. *p < 0.05, **p < 0.01, ***p < 0.001. FAS, full analysis set; LS, least squares; MMRM, mixed models for repeated measures; NPI, Neuropsychiatric Inventory; NPI‐Distress, Neuropsychiatric Inventory‐Distress; SE, standard error.

For NPI total score, the means (SD) at baseline in the placebo, brexpiprazole 1 mg, and 2 mg groups were 38.1 (17.4), 36.8 (17.3), and 37.3 (19.1), respectively. The least squares (LS) mean changes (standard error [SE]) from baseline to Week 10 were −8.8 (1.42), −10.0 (1.67), and −17.3 (1.47; Figure 1), and the differences of brexpiprazole 1 and 2 mg versus placebo were −1.2 (95% confidence interval [CI]: −5.4, 3.1, *p* = 0.5891, SMD: 0.070) and −8.4 (95% CI: −12.4, −4.5, *p* < 0.0001, SMD: 0.485), respectively.

**FIGURE 2:**
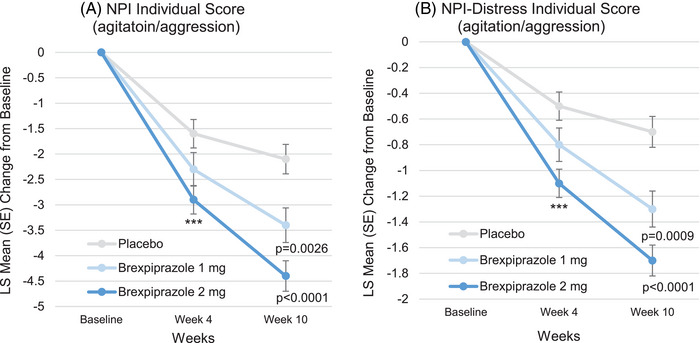
Time course of NPI and NPI‐Distress individual scores (agitation/aggression) (FAS). MMRM analysis. ***p < 0.001. FAS, full analysis set; LS, least squares; MMRM, mixed models for repeated measures; NPI, Neuropsychiatric Inventory; NPI‐Distress, Neuropsychiatric Inventory‐Distress; SE, standard error.

**FIGURE 3:**
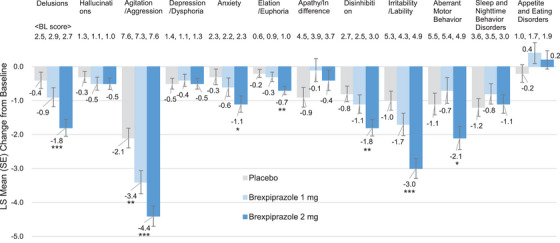
Changes in NPI individual scores from baseline to Week 10 (FAS). MMRM analysis. *p < 0.05, **p < 0.01, ***p < 0.001. BL, baseline; FAS, full analysis set; LS, least squares; MMRM, mixed models for repeated measures; NPI, Neuropsychiatric Inventory; SE, standard error.

For NPI individual scores, the means (SD) of agitation/aggression score at baseline in the placebo, brexpiprazole 1 mg, and 2 mg groups were 7.6 (2.5), 7.3 (2.6), and 7.6 (2.6), respectively. The LS mean changes (SE) from baseline to Week 10 were −2.1 (0.29), −3.4 (0.34), and −4.4 (0.30; Figure 2), and the differences of brexpiprazole 1 and 2 mg versus placebo were −1.3 (95% CI: −2.2, −0.5, *p* = 0.0026, SMD: 0.380) and −2.3 (95% CI: −3.1, −1.5, *p* < 0.0001, SMD: 0.656), respectively. In relation to agitation/aggression, the means (SD) of irritability/lability score at baseline in the placebo, brexpiprazole 1 mg, and 2 mg groups were 5.3 (4.0), 4.3 (4.0), and 4.9 (4.0), respectively. The LS mean changes (SE) from baseline to Week 10 were −1.0 (0.28), −1.7 (0.33), and −3.0 (0.29; Figure 3), and the differences of brexpiprazole 1 and 2 mg versus placebo were −0.7 (95% CI: −1.5, 0.2, *P *= 0.1228, SMD: 0.205) and −1.9 (95% CI: −2.7, −1.1, *p* < 0.0001, SMD: 0.542), respectively. In addition, ≥ 1 point improvement in the difference of brexpiprazole 2 mg versus placebo was also observed in other individual scores: the difference of delusions was −1.4 (95% CI: −2.0, −0.7, *p* < 0.0001, SMD: 0.492), that of disinhibition was −1.0 (95% CI: −1.7, −0.4, *p* = 0.0015, SMD: 0.351), and that of aberrant motor behavior scores was −1.0 (95% CI: −1.9, −0.1, *P *= 0.0235, SMD: 0.254).

For NPI‐Distress total score, the means (SD) at baseline in the placebo, brexpiprazole 1 mg, and 2 mg groups were 16.0 (7.5), 14.9 (7.5), and 15.8 (8.1), respectively. The LS mean changes (SE) from baseline to Week 10 were −3.4 (0.59), −4.5 (0.70), and −7.3 (0.62; Figure 1), and the differences of brexpiprazole 1 and 2 mg versus placebo were −1.1 (95% CI: −2.9, 0.7, *P *= 0.2292, SMD: 0.152) and −3.9 (95% CI: −5.6, −2.3, *p* < 0.0001, SMD: 0.539), respectively.

**FIGURE 4:**
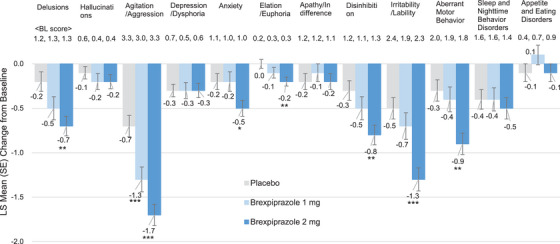
Changes in NPI‐Distress individual scores from baseline to Week 10 (FAS). MMRM analysis. *p < 0.05, **p < 0.01, ***p < 0.001. BL, baseline; FAS, full analysis set; LS, least squares; MMRM, mixed models for repeated measures; NPI‐Distress, Neuropsychiatric Inventory Distress; SE, standard error.

For NPI‐Distress individual scores, the means (SD) of agitation/aggression score at baseline in the placebo, brexpiprazole 1 mg, and 2 mg groups were 3.3 (1.0), 3.0 (1.0), and 3.3 (0.9), respectively. The LS mean changes (SE) from baseline to Week 10 were −0.7 (0.12), −1.3 (0.14), and −1.7 (0.12; Figure 2), and the differences of brexpiprazole 1 and 2 mg versus placebo were −0.6 (95% CI: −1.0, −0.2, *P *= 0.0009, SMD: 0.373) and −1.0 (95% CI: −1.4, −0.7, *p* < 0.0001, SMD: 0.652), respectively. In relation to agitation/aggression, the means (SD) of irritability/lability score at baseline in the placebo, brexpiprazole 1 mg, and 2 mg groups were 2.4 (1.6), 1.9 (1.6), and 2.3 (1.7), respectively. The LS mean changes (SE) from baseline to Week 10 were −0.5 (0.12), −0.7 (0.15), and −1.3 (0.13; Figure 4), and the differences of brexpiprazole 1 and 2 mg versus placebo were −0.2 (95% CI: −0.6, 0.2, *p* = 0.2857, SMD: 0.124) and −0.8 (95% CI: −1.2, −0.5, *p* < 0.0001, SMD: 0.522), respectively.

For the timing of improvements, we analyzed Week 4 as well as Week 10 (Figures 1 and 2, and Figure S1 and S2 in supporting information). In the NPI and NPI‐Distress total scores, the improvements were observed as early as Week 4, but the extent of improvements was modest compared to that of Week 10. In the NPI total score, the differences of brexpiprazole 2 mg versus placebo at Week 4 and Week 10 were −5.7 (95% CI: −9.0, −2.4, *p* = 0.0007, SMD: 0.394) and −8.4 (95% CI: −12.4, −4.5, *p* < 0.0001, SMD: 0.485), respectively. In NPI‐Distress total score, the differences of brexpiprazole 2 mg versus placebo at Week 4 and Week 10 were −2.2 (95% CI: −3.6, −0.8, *P =* 0.0018, SMD: 0.359) and −3.9 (95% CI: −5.6, −2.3, *p* < 0.0001, SMD: 0.539), respectively. Enhancing tendency from Week 4 to Week 10 was also observed in NPI and NPI‐Distress individual score of agitation/aggression. In NPI agitation/aggression, the differences of brexpiprazole 2 mg versus placebo at Week 4 and Week 10 were −1.3 (95% CI: −2.0, −0.5, *p* = 0.0008, SMD: 0.396) and −2.3 (95% CI: −3.1, −1.5, *p* < 0.0001, SMD: 0.656), respectively. In NPI‐Distress agitation/aggression, the differences of brexpiprazole 2 mg versus placebo at Week 4 and Week 10 were −0.6 (95% CI: −0.9, −0.3, *p* < 0.0001, SMD: 0.456) and −1.0 (95% CI: −1.4, −0.7, *p* < 0.0001, SMD: 0.652), respectively. These tendencies were also observed in NPI and NPI‐Distress individual scores of delusions, disinhibition, irritability/lability, and aberrant motor behavior. For raw scores of time course of NPI and NPI‐Distress total scores, and NPI and NPI‐Distress individual scores, see Figures S3–S15 in supporting information.

### 3.3 Other findings

The results of primary efficacy and safety endpoints were reported previously.^20^ Briefly, for the change in CMAI total score from baseline to Week 10 (primary endpoint), both brexpiprazole 1 and 2 mg groups demonstrated statistically significant improvement versus placebo (2 mg: LS mean difference –7.2 [95% CI: –10.0, –4.3, *p* < 0.0001], 1 mg: LS mean difference –3.7 [95% CI: –6.8, –0.7, *p* = 0.0175]). The incidences of treatment‐emergent adverse events reported in the placebo, brexpiprazole 1 mg, and 2 mg groups were 73.8%, 76.8%, and 84.6%, respectively.

## 4 DISCUSSION

The efficacy and safety of brexpiprazole for AAD in the Japanese and international studies have already been reported.^20^, ^21^, ^22^, ^23^, ^24^ When it comes to NPI, however, the results of a post hoc pooled analysis of the two international pivotal studies were presented only in a limited way at the American Association for Geriatric Psychiatry 2024 annual meeting,^25^ and the data are not yet published in the form of an article. In this article, we have analyzed the exploratory endpoints of the Japanese pivotal study and have reported the improvements of both NPI and NPI‐Distress scores by 10 week brexpiprazole treatment. These results suggest that brexpiprazole improves patient BPSD and reduces caregiver distress. To our knowledge, this article is the first report of the brexpiprazole's impact on both patient neuropsychiatric symptoms by NPI individual scores and caregiver distress by NPI‐Distress total and individual scores. In addition, before our study, atypical antipsychotics (brexpiprazole falls in this category) have never been assessed for NPI and NPI‐Distress in the pivotal study of Japanese patients.

For NPI total score, both the brexpiprazole 1 and 2 mg groups showed greater improvement from baseline at Week 10 compared to the placebo group, and the 2 mg group (difference vs. placebo: −8.4, *p* < 0.0001, SMD: 0.485) had greater improvement than the 1 mg group (difference vs. placebo: −1.2, *p* = 0.5891, SMD: 0.070). As for a minimum clinically important difference (MCID) for NPI total score, a couple of reports have been published previously,^38^, ^39^ and the latest one by Howard et al. has reported the MCID as 8.0.^40^ Although there is no generally accepted MCID for NPI, using this MCID of 8.0, the brexpiprazole 2 mg group demonstrated the comparable level of efficacy at Week 10, that is, the difference versus the placebo group was –8.4 (*p* < 0.0001, SMD: 0.485). Similar results were observed in agitation/aggression score, that is, the 2 mg group (difference vs. placebo: −2.3, *p* < 0.0001, SMD: 0.656) had greater improvement than the 1 mg group (difference vs. placebo: −1.3, *p* = 0.0026, SMD: 0.380) at Week 10. These results were consistent with and supported the result of the primary endpoint CMAI reported previously.^20^ In addition, the brexpiprazole 2 mg group showed greater improvements in a diverse range of individual scores versus the placebo group at Week 10, for example, not only agitation/aggression score, but also delusions, disinhibition, irritability/lability, and aberrant motor behavior scores (≥ 1 point improvement), of which agitation/aggression related irritability/lability showed approximately 2.0 points improvement (difference vs. placebo: −1.9, *p* < 0.0001, SMD: 0.542). These results suggest that the improvements in the NPI total score were mainly derived from the improvements of these individual scores, and that brexpiprazole is beneficial to improve various BPSD associated with Alzheimer's dementia.

For NPI‐Distress total score, similar results were observed. At Week 10, the 2 mg group (difference vs. placebo: −3.9, *p* < 0.0001, SMD: 0.539) had greater improvement than the 1 mg group (difference vs. placebo: −1.1, *P *= 0.2292, SMD: 0.152). In agitation/aggression score, the 2 mg group (difference vs. placebo: −1.0, *p* < 0.0001, SMD: 0.652) had greater improvement than the 1 mg group (difference vs. placebo: −0.6, *p* = 0.0009, SMD: 0.373). These results indicate brexpiprazole is also beneficial to reduce caregiver distress. In terms of NPI‐Distress individual scores, the baseline score of agitation/aggression was approximately 3 points, those of irritability/lability and aberrant motor behavior were approximately 2 points, and many of the other domains were approximately ≤ 1 point. In accordance with this environment, the brexpiprazole 2 mg group showed greater improvement (≥ 1 point improvement) in agitation/aggression score and modest improvement (< 1 point improvement) in delusions, disinhibition, irritability/lability, and aberrant motor behavior scores versus the placebo group.

For the timing of improvements, the data were collected at baseline, Week 4, and Week 10, and analyzed accordingly. In the 2 mg group, the improvement of NPI total score was observed as early as Week 4, but the extent of improvement was less than MCID 8.0 points (difference vs. placebo: −5.7, *p* = 0.0007, SMD: 0.394). The MCID level improvement was observed at Week 10 (difference vs. placebo: −8.4, *p* < 0.0001, SMD: 0.485). In the 1 mg group, the MCID level improvement was not observed at both Week 4 and Week 10. These results suggest that brexpiprazole 2 mg treatment for 10 weeks is expected to achieve the MCID level improvement of NPI total score.

In comparison to the other studies, similar results were observed from a post hoc pooled analysis of the two international pivotal studies,^25^ in which the NPI total score, NPI agitation/aggression score, NPI‐Distress total score, and NPI‐Distress agitation/aggression score were all improved. This post hoc pooled analysis also analyzed NPI‐4A subscale (agitation/aggression, irritability/lability, aberrant motor behavior, and anxiety domains) and NPI‐4D subscale (agitation/aggression, irritability/lability, aberrant motor behavior, and disinhibition domains), and both subscales were improved as well. Although direct comparison is impossible due to different study designs, these results are consistent with those of our study.

Sex distribution in this study was a little uneven. Sometimes this happens in a clinical study incidentally. We consider the differences observed in this study were not significant enough to affect the results.

Limitations of our analyses are as follows. NPI and NPI‐Distress were completed by investigators, based on information from caregivers (to minimize the influences caused by different caregivers, the protocol defined the requirements for setting a primary caregiver, such as available at least 4 days/week and 4 hours/day for patient observation, etc.). In both of NPI and NPI‐Distress scores, the individual scores at baseline vary from < 1.0 to > 7.0. In the case of lower scores, it is unknown whether brexpiprazole had no efficacy or if there was no room for improvement. As the endpoints of NPI and NPI‐Distress were exploratory ones, statistical analyses were conducted in a non‐confirmatory manner and the results were presented descriptively. For the missing mechanism, we hypothesized missing at random, but if this hypothesis was not valid, the results could be biased. Among various background factors, such as age, sex, medical care category (inpatient or outpatient [home or care facility]), prior use of antipsychotics, and so on, there may be factors that influence efficacy results as confounders. For NPI and NPI‐Distress, changes in the total and individual scores from baseline were evaluated up to Week 10, and thus the results over a longer period are unknown. The recurrence of symptoms and its impact on caregiver distress after the cessation of brexpiprazole treatment also remain unknown. This study was conducted in Japanese patients, and therefore caution is necessary when extrapolating the results to other races.

In conclusion, our results suggest that brexpiprazole treatment for AAD is beneficial to improve patient BPSD and to reduce caregiver distress.

## CONFLICT OF INTEREST STATEMENT

Yu Nakamura has received speakers’ honoraria, manuscript fee, research support, or scholarship donation from Otsuka Pharmaceutical Co., Ltd., Meiji Seika Pharma Co., Ltd., Viatris Pharmaceutical K.K., Eisai Co., Ltd., Takeda Pharmaceutical Co., Ltd., Teikoku Pharmaceutical K.K., Kowa Company Ltd., Mochida Pharmaceutical Co., Ltd., Towa Pharmaceutical Co., Ltd., MSD K.K., Biogen Japan Ltd., Daiichi Sankyo Company Ltd, and EA Pharma Co., Ltd. Jun Adachi, Naoki Hirota, Katsuhiro Iba, Cosmo Sasajima, Koichi Shimizu, Masami Nakai, and Kaneyoshi Takahashi are full‐time employees of Otsuka Pharmaceutical Co., Ltd. Author disclosures are available in the supporting information.

## CONSENT STATEMENT

All patients provided written informed consent.

## Supporting information



Supporting Information

Supporting Information
