# Supplementary material for: Brexpiprazole's impacts on patients and caregivers in agitation in Alzheimer's dementia
Source: Alzheimers Dement. 2025 Jul 28;21(7):e70522. doi: 10.1002/alz.70522 (PMC12301695; doi:10.1002/alz.70522)
Supplement: Supplementary file 1 — Supporting Information [file ALZ-21-e70522-s001.pdf]

Supplemental Figure 1. Changes in NPI Individual Scores from Baseline to Week 4 (FAS)

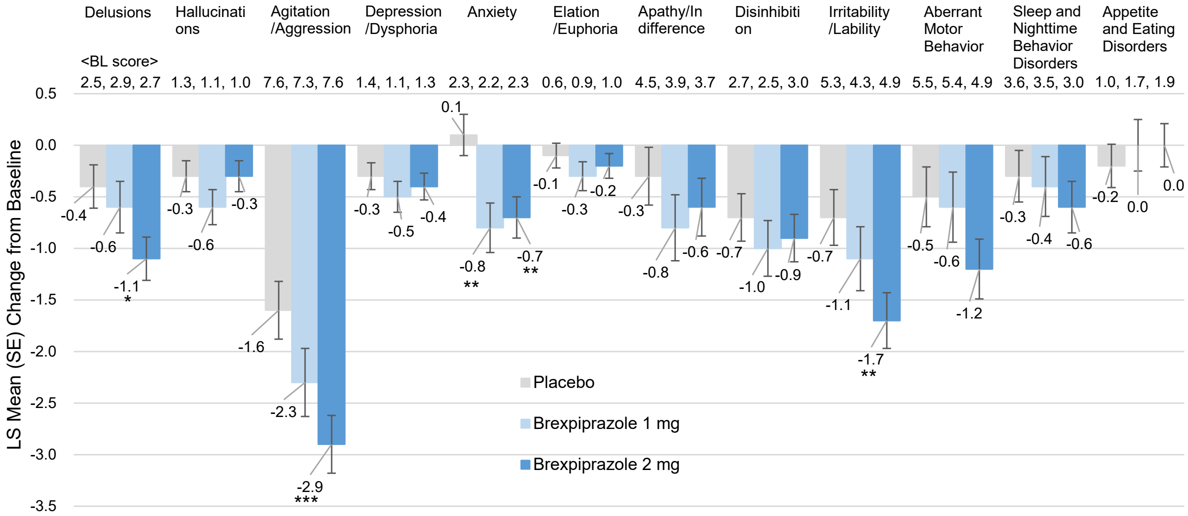

MMRM analysis. BL, baseline; FAS, full analysis set; LS, least squares; MMRM, mixed models for repeated measures; NPI, Neuropsychiatric Inventory; SE, standard error. *p<0.05, **p<0.01, ***p<0.001.

Supplemental Figure 2. Changes in NPI-Distress Individual Scores from Baseline to Week 4 (FAS)

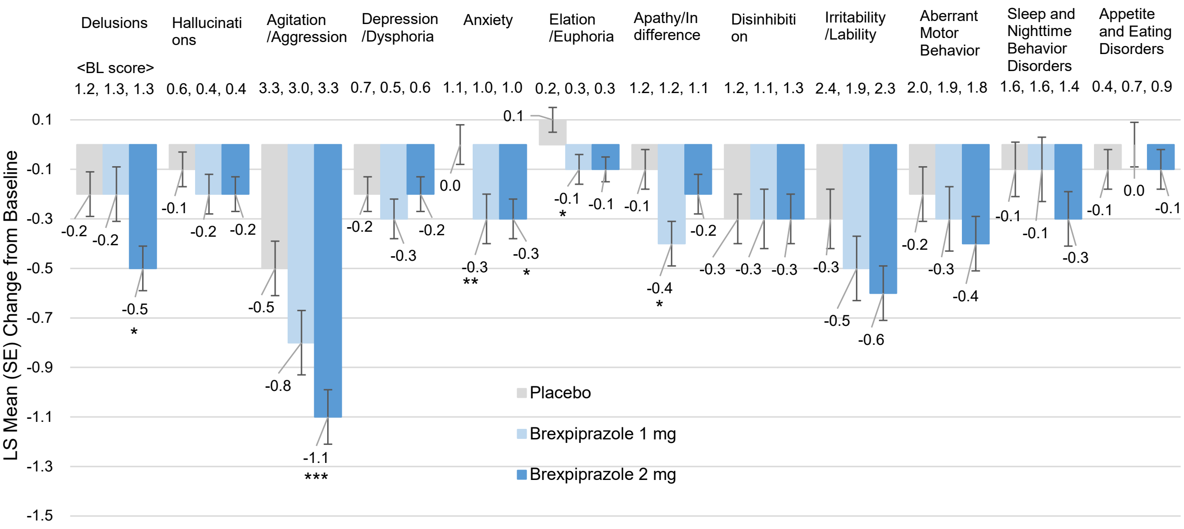

MMRM analysis. BL, baseline; FAS, full analysis set; LS, least squares; MMRM, mixed models for repeated measures; NPI-Distress, Neuropsychiatric Inventory - Distress; SE, standard error. *p<0.05, **p<0.01, ***p<0.001.

Supplemental Figure 3. Time Course of NPI and NPI-Distress Total Scores (FAS)

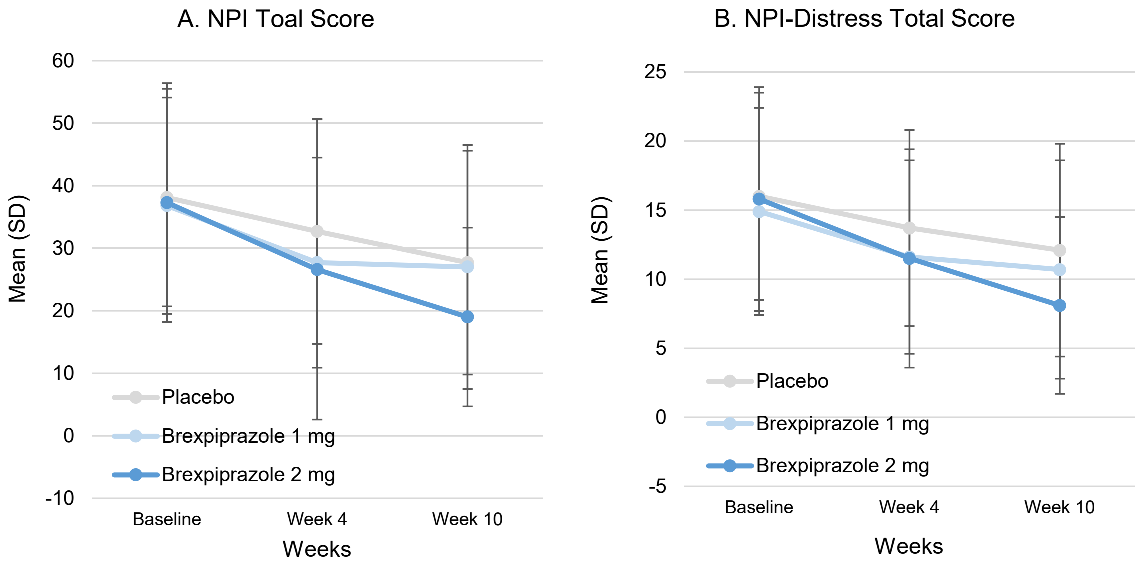

MMRM analysis. FAS, full analysis set; MMRM, mixed models for repeated measures; NPI, Neuropsychiatric Inventory; NPI-Distress, Neuropsychiatric Inventory - Distress; SD, standard deviation.

Supplemental Figure 4. Time Course of NPI and NPI-Distress Individual Scores (agitation/aggression) (FAS)

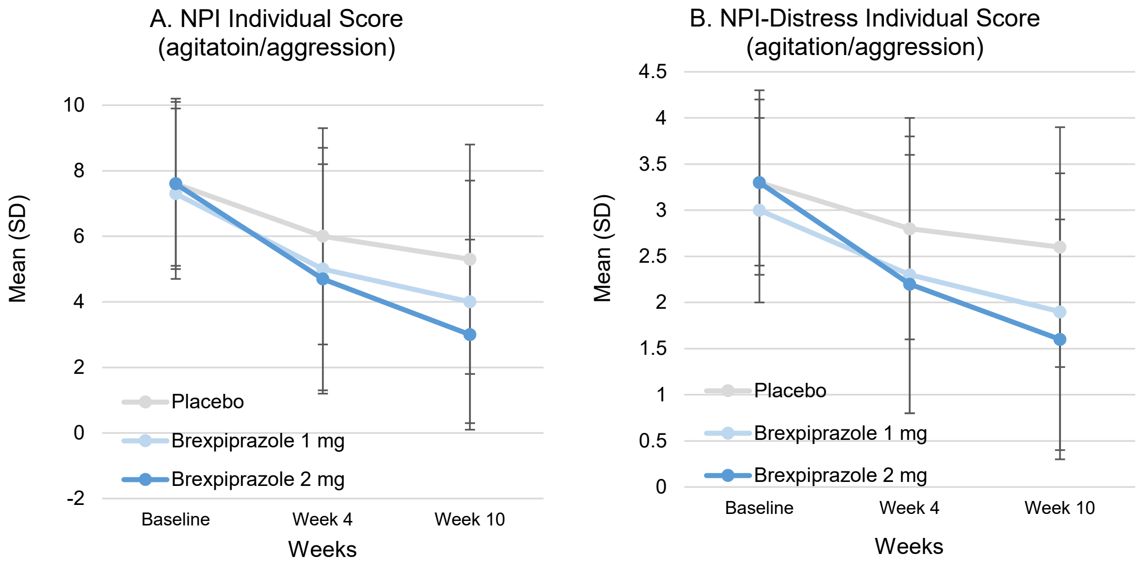

MMRM analysis. FAS, full analysis set; MMRM, mixed models for repeated measures; NPI, Neuropsychiatric Inventory; NPI-Distress, Neuropsychiatric Inventory - Distress; SD, standard deviation.

Supplemental Figure 5. Time Course of NPI and NPI-Distress Individual Scores (Delusions) (FAS)

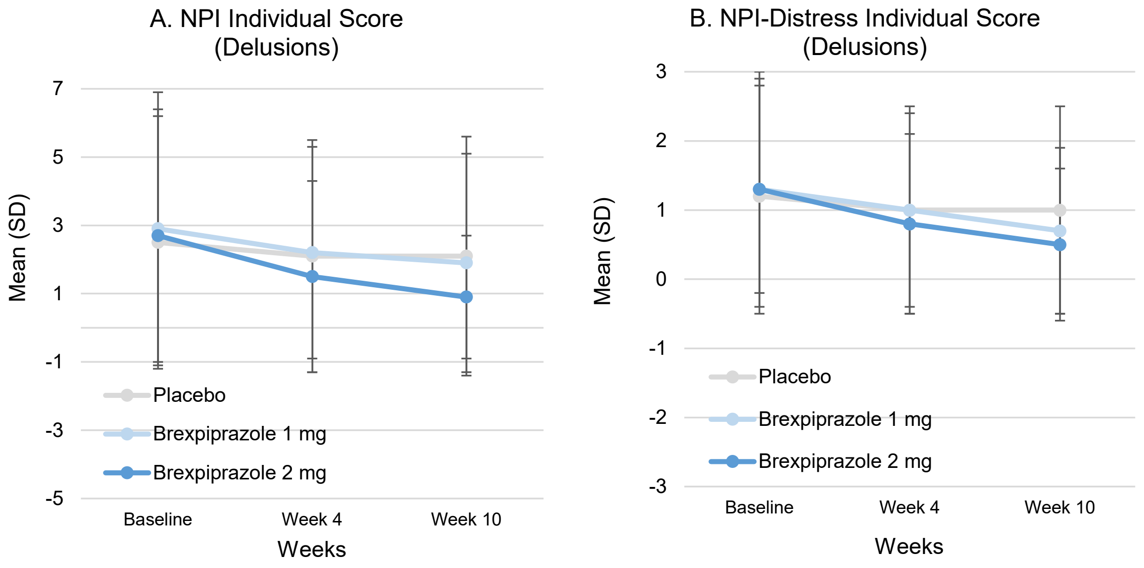

MMRM analysis. FAS, full analysis set; MMRM, mixed models for repeated measures; NPI, Neuropsychiatric Inventory; NPI-Distress, Neuropsychiatric Inventory - Distress; SD, standard deviation.

Supplemental Figure 6. Time Course of NPI and NPI-Distress Individual Scores (Hallucinations) (FAS)

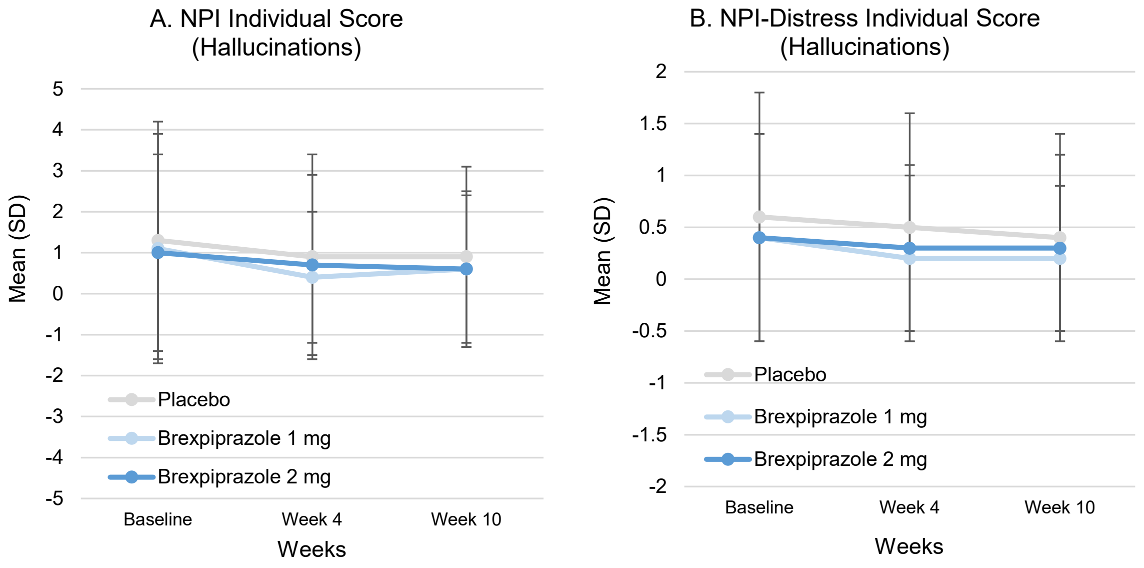

MMRM analysis. FAS, full analysis set; MMRM, mixed models for repeated measures; NPI, Neuropsychiatric Inventory; NPI-Distress, Neuropsychiatric Inventory - Distress; SD, standard deviation.

Supplemental Figure 7. Time Course of NPI and NPI-Distress Individual Scores (Depression/Dysphoria) (FAS)

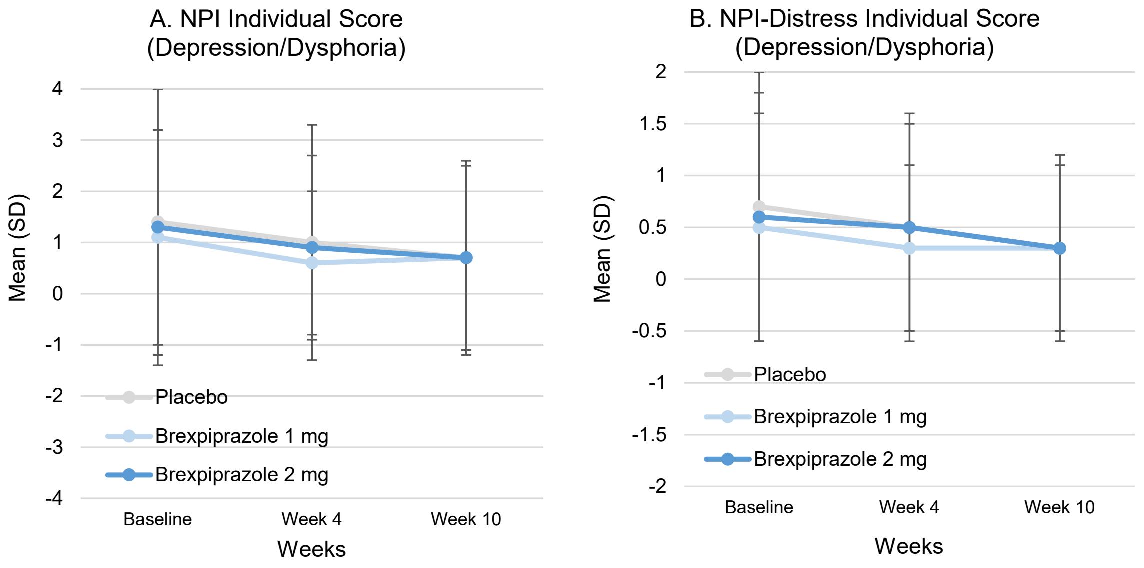

MMRM analysis. FAS, full analysis set; MMRM, mixed models for repeated measures; NPI, Neuropsychiatric Inventory; NPI-Distress, Neuropsychiatric Inventory - Distress; SD, standard deviation.

Supplemental Figure 8. Time Course of NPI and NPI-Distress Individual Scores (Anxiety) (FAS)

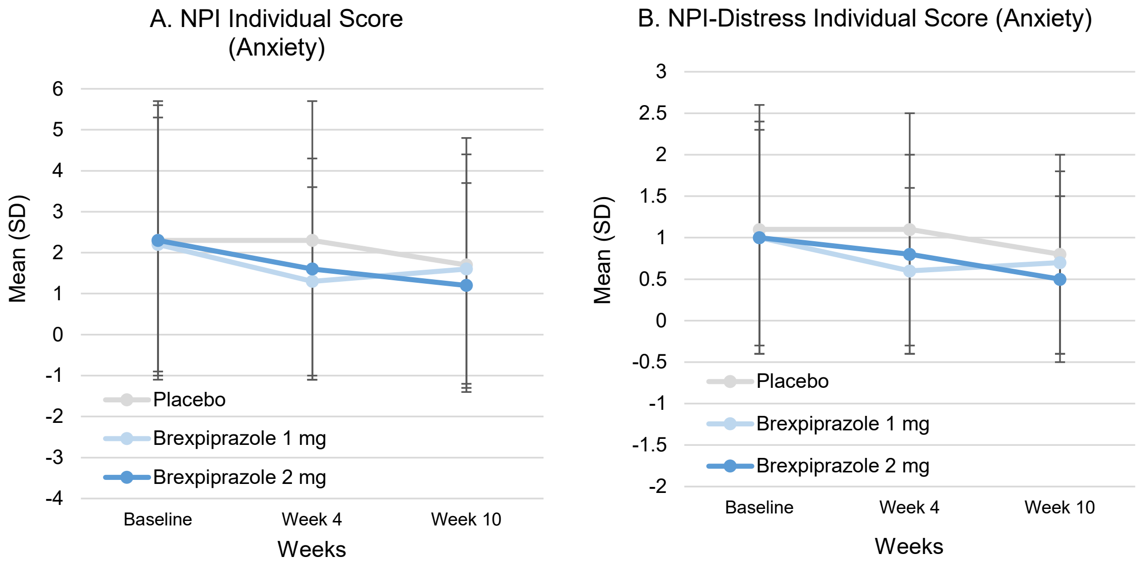

MMRM analysis. FAS, full analysis set; MMRM, mixed models for repeated measures; NPI, Neuropsychiatric Inventory; NPI-Distress, Neuropsychiatric Inventory - Distress; SD, standard deviation.

Supplemental Figure 9. Time Course of NPI and NPI-Distress Individual Scores (Elation/Euphoria) (FAS)

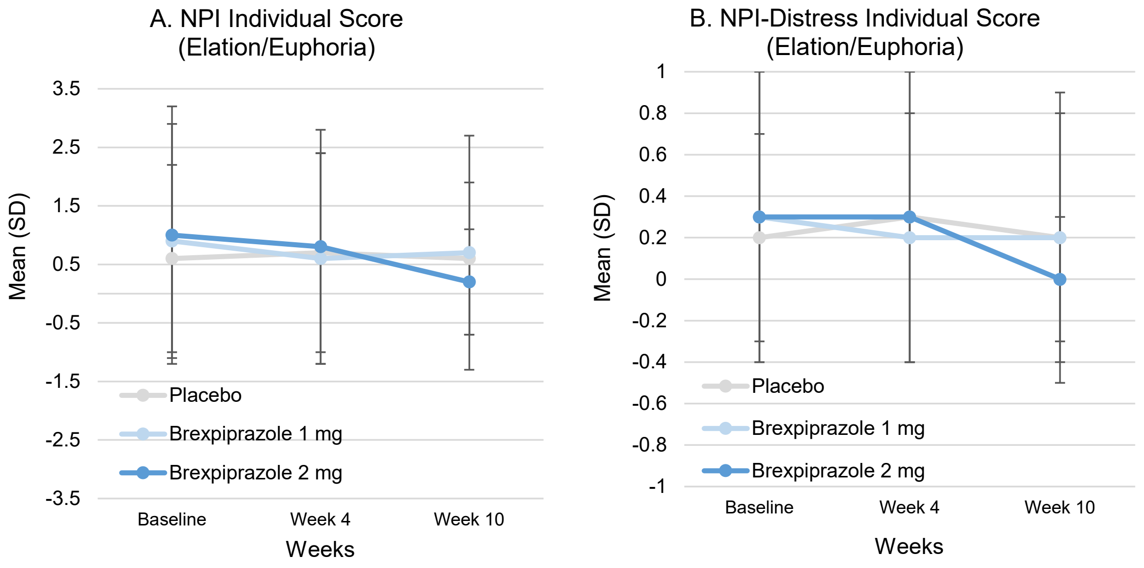

MMRM analysis. FAS, full analysis set; MMRM, mixed models for repeated measures; NPI, Neuropsychiatric Inventory; NPI-Distress, Neuropsychiatric Inventory - Distress; SD, standard deviation.

Supplemental Figure 10. Time Course of NPI and NPI-Distress Individual Scores (Apathy/Indifference) (FAS)

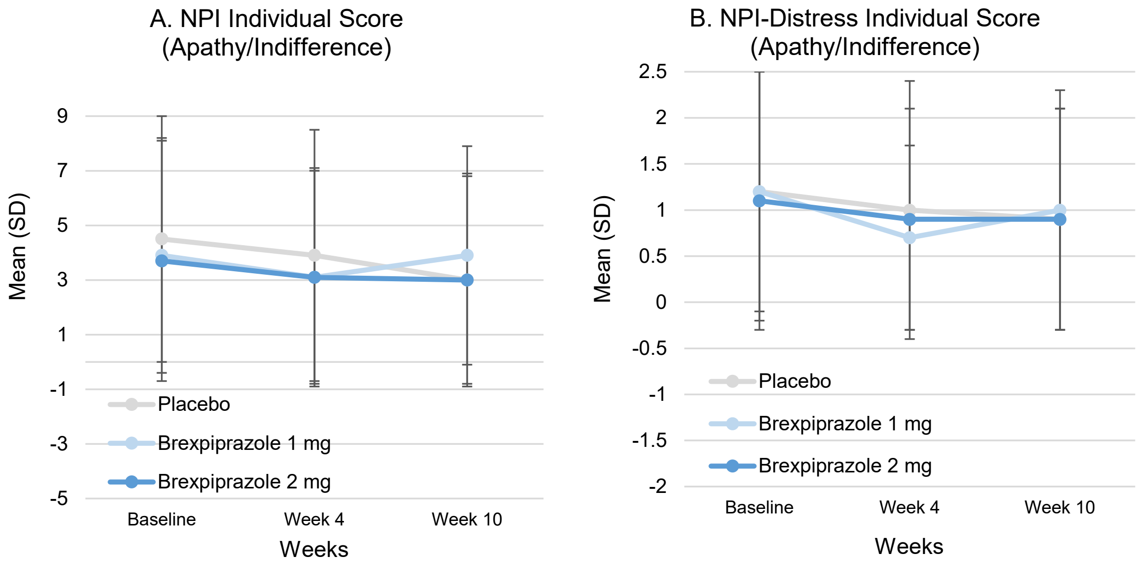

MMRM analysis. FAS, full analysis set; MMRM, mixed models for repeated measures; NPI, Neuropsychiatric Inventory; NPI-Distress, Neuropsychiatric Inventory - Distress; SD, standard deviation.

Supplemental Figure 11. Time Course of NPI and NPI-Distress Individual Scores (Disinhibition) (FAS)

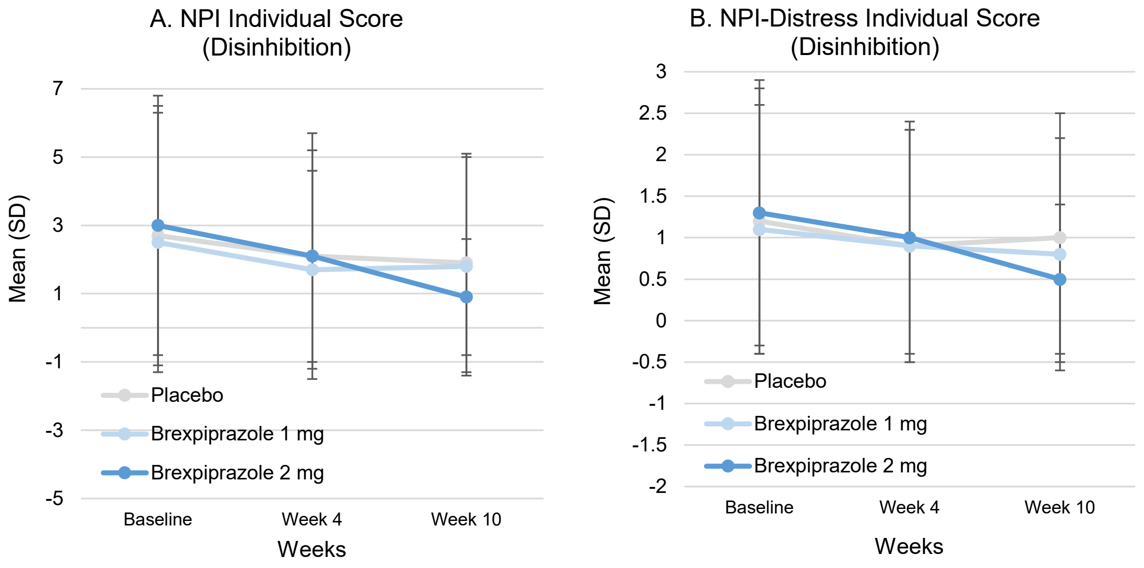

MMRM analysis. FAS, full analysis set; MMRM, mixed models for repeated measures; NPI, Neuropsychiatric Inventory; NPI-Distress, Neuropsychiatric Inventory - Distress; SD, standard deviation.

Supplemental Figure 12. Time Course of NPI and NPI-Distress Individual Scores (Irritability/Lability) (FAS)

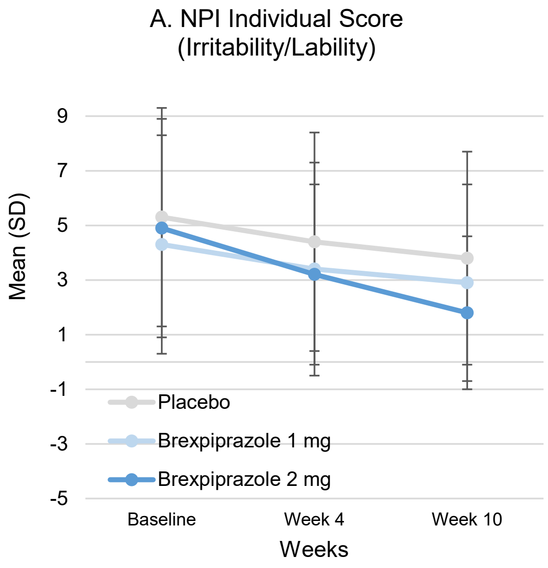

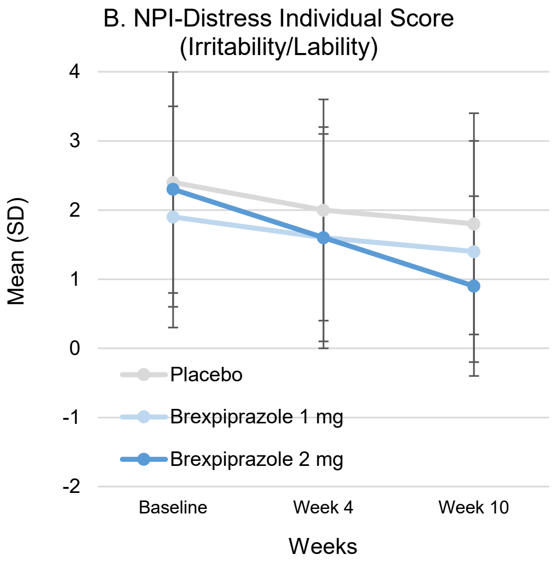

MMRM analysis. FAS, full analysis set; MMRM, mixed models for repeated measures; NPI, Neuropsychiatric Inventory; NPI-Distress, Neuropsychiatric Inventory - Distress; SD, standard deviation.

Supplemental Figure 13. Time Course of NPI and NPI-Distress Individual Scores (Aberrant Motor Behavior) (FAS)

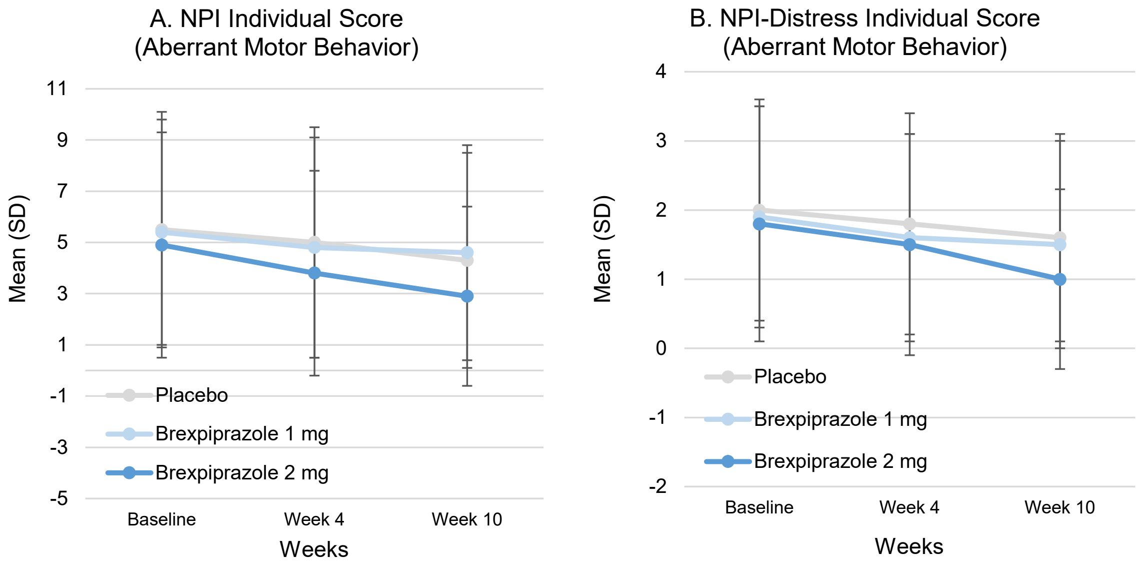

MMRM analysis. FAS, full analysis set; MMRM, mixed models for repeated measures; NPI, Neuropsychiatric Inventory; NPI-Distress, Neuropsychiatric Inventory - Distress; SD, standard deviation.

Supplemental Figure 14. Time Course of NPI and NPI-Distress Individual Scores (Sleep and Nighttime Behavior Disorders) (FAS)

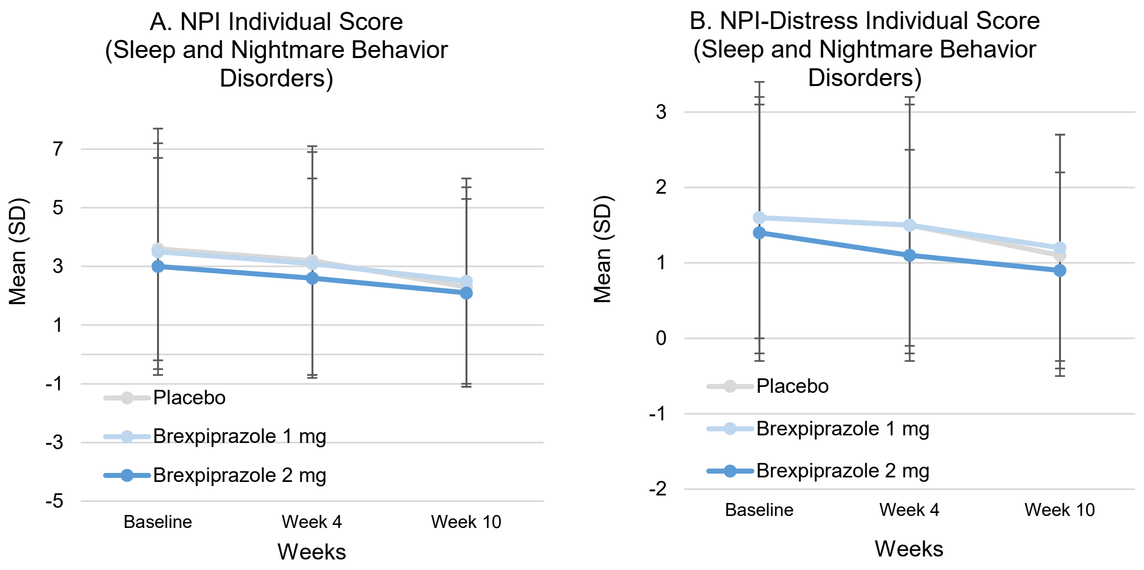

MMRM analysis. FAS, full analysis set; MMRM, mixed models for repeated measures; NPI, Neuropsychiatric Inventory; NPI-Distress, Neuropsychiatric Inventory - Distress; SD, standard deviation.

Supplemental Figure 15. Time Course of NPI and NPI-Distress Individual Scores (Appetite and Eating Disorders) (FAS)

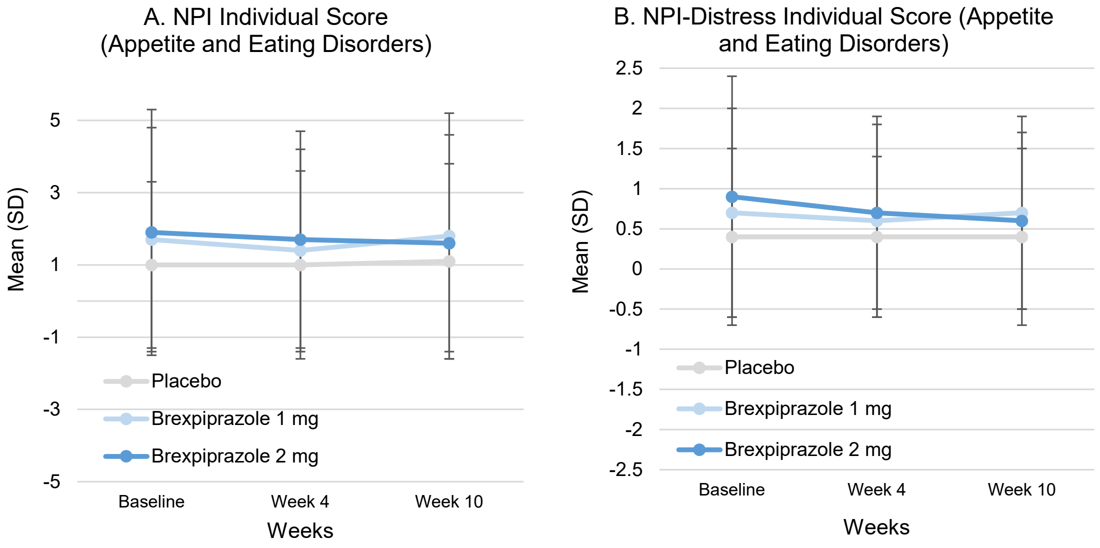

MMRM analysis. FAS, full analysis set; MMRM, mixed models for repeated measures; NPI, Neuropsychiatric Inventory; NPI-Distress, Neuropsychiatric Inventory - Distress; SD, standard deviation.
